# Scientific Items

**Published:** 1878-01-20

**Authors:** 


### *SCIENTIFIC ITEMS.*

### EDISON'S PHONOGRAPH.

Mr. Thomas Edison, of Menlo Park, N. J., is the inventor of the Phonograph, an instrument by which the vibration of sound causes indentations upon lead paper moving upon a cylinder. "This cylinder, from one end of it to the other, has small lines cut into it and running around it. By the side of the cylinder, and resting on it, is a pin attached to a vibrating plate, over which is a mouthpiece with a small hole leading to the plate immediately over the hole. When the phonograph is to be used a sheet of lead paper is spread over the cylinder, the words or sounds to be reproduced are made very close to the mouthpiece, and the vibration in the air occasioned by their utterance cause the plate to move, and the pin indents the lead paper upon the cylinder, which, by the way, is turned by means of the crank duiing the operation. The words being spoken, the paper is indented by the pin and the crank turns the cylinder back to its starting point. A funnel is then placed over the mouthpiece. Mr. Edison used one made of a plain piece of writing paper. The cylinder is then turned over the same space it originally traversed, and the sounds are reproduced accurately, and can be heard for several feet around the instrument, varying always with the loudness of the tone in which the words were spoken to the machine. After receiving the indentations made by the pin, the lead paper can be taken off the cylinder, and by replacing it at any other time the words can be exactly reproduced. This is what Mr. Edison thinks will make the machine practically valuable.

When the reporter of the *World* entered the room in which the exhibition was given, Mr. Edison was singing to the machine "Tommy, Make Room for Your Uncle." The reporter expected when the funnel was placed over the mouthpiece to only hear inarticulate words reproduced, but he was astonished in hearing the words of the song quite as plainly as he heard them whem uttered by Mr. Edison. Mr. Edison tried the effect of turning the crank slowly, and the slower it moved the deeper was the sound of the reproduction. As the crank was moved faster they came again in a shrill tenor.

Several days ago Mr. Edison adjusted the phonograph to the telephone, and succeeded perfectly in sending a conversation over the telegraph wires from his laborator at Merlo Park to Newark, a distance of seven miles.—*Exchange.*

---

In the *Jewish Talmud* there are evidences of a much more advanced knowledge of the sciences in ancient times than we are wont to believe. It is related of Rabbi Gamalial that he had in his house a kind of orrery for studying the motions of the planets; this as early as the 30th year of the common Era. It is also stated that he had a tube by means of which he could observe objects at great distances—answering to our telescope.

Some sort of anæsthetic agent was also known, as the following quotation occurs: "They gave him to drink a potion, which cast him into a profound sleep so that they were enabled to perform the operation of gastronomy." Something is also said about artificial teeth, and a "tooth covered with gold so as to stop and hide the decay."

---

### SUN'S DISTANCE.

The distance to the sun, as estimated by parties sent out by the British authorities to make observations of the transit of Venus, is 93,375,000 miles.

---

It is said that electrical wires around a tin can are capable of transmitting telephonic sounds to another can at the distance of many miles.

